# Pitfalls in the location of guest molecules in metal-organic frameworks

**DOI:** 10.1038/s41467-022-32890-0

**Published:** 2022-09-09

**Authors:** Tomasz Poręba, Piero Macchi, Michelle Ernst

**Affiliations:** 1 European Synchrotron Radiation Facility, 71 Avenue des Martyrs, 38000 Grenoble, France; 2 Department of Chemistry, Materials and Chemical Engineering, Polytechnic of Milan, Via Mancinelli 7, 20131 Milan, Italy; 3 Computational Carbon Chemistry Group, Heidelberg Institute for Theoretical Studies, 69118 Heidelberg, Germany; 4 Interdisciplinary Center for Scientific Computing, Heidelberg University, 69120 Heidelberg, Germany

**Keywords:** Metal-organic frameworks, Characterization and analytical techniques, Sensors

**arising from** B. Wang et al. *Nature Communications* 10.1038/s41467-019-11912-4 (2019)

Recently, Wang B. et al. reported a zirconium metal-organic framework (MOF) with high sensing abilities towards polychlorinated dibenzo-*p*-dioxins^1^. The sensing mechanism is based on fluorescence quenching from the dioxin-loaded MOF. The structure of the framework with the absorbed guest molecules (2,3-dichlorodibenzo-p-dioxin, BCDD) has been determined via single-crystal X-ray diffraction (XRD). Based on these measurements, the authors discuss the host-guest interactions. The quality of the diffraction data, however, does not allow to reliably determine the presence of BCDD in the pores.

Wang B. et al. recently reported the synthesis and structural characterisation of a new MOF (BUT-17) that they claimed capable of incorporating polychlorinated dioxins. To prove this, they immersed the single-crystals of the guest-free BUT-17 in a hexane solution of BCDD, and left the solvent to slowly evaporate. Subsequently, they mounted, on a goniometer head, a single-crystal of the MOF with incorporated BCDD and flushed with nitrogen at 50 °C for 5 h to remove the residuary solvent. Then, they collected XRD data at 100 K, and reported the structural model of BCDD@BUT-17 (refcode: JOMZOD in the Cambridge Structural Database). The authors positioned the BCDD molecules at the borders of the large hexagonal channels, bound to the framework through the cooperation of π-π stacking, hydrogen bonding and “molecular clipping” with the formate groups.

After a first inspection of the structure, one is immediately impressed by the unusually large displacement parameters (U_iso_) of the BCDD atoms, despite a partial occupation of only 16%, fixed without any justification. Moreover, the model does not consider the inherent disorder due to the molecule sitting about an mm2 symmetry site. Thus, the modelled molecule is actually tetra-chlorodibenzo-*p*-dioxin instead of BCDD.

Based on the reported XRD data, we refined the structural model fixing the U_iso_ to those refined for the MOF organic linkers (0.04(1) Å^2^ on average). The resulting occupancies drop to 1% or smaller except for C17 and C18, which have site occupation factors of ca. 8 and 3%, respectively. This clearly shows that the electron density peaks do not form a connected set of the modelled molecule. Instead, the geometry of the BCDD molecule was forced by applying a number of restraints on the interatomic distances, the molecular conformation, and the displacement parameters^1^. Without those restraints, the molecular geometry collapses during a refinement, suggesting that there is not enough experimental evidence of its presence.

The accuracy of the model can be easily evaluated by the Fourier maps that we computed from the deposited diffraction data. We used the same software adopted by the authors (Shelxl^2^ implemented in Olex2^3^) and assigned the phases of the structure factors from the reported model.

The map of the total electron density in the plane of BCDD (Fourier transform of F_obs_) reveals no maximum corresponding to atoms of the molecule included in the model (Fig. 1a). For comparison, Fig. 1b shows the same map in the region of the organic linker, which instead clearly stands out.

**Fig. 1:**
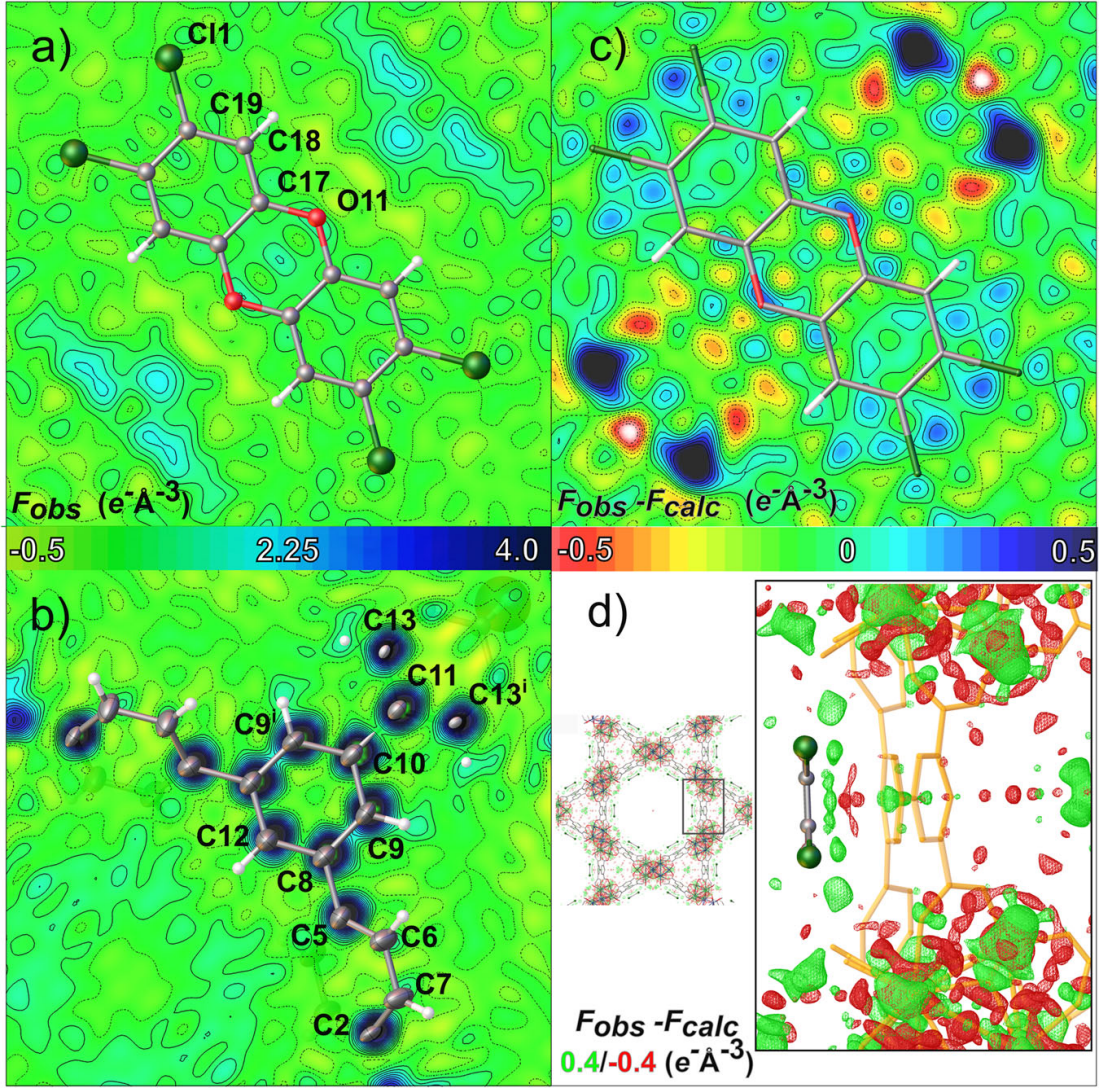
Maps of the total electron density of BCDD@BUT-17 (in eÅ^−3^ units, see the colour scale bars) calculated from the deposited structure factors. F_obs_ maps plotted in the plane where BCDD was positioned (**a**) and in the plane of the organic linker (**b**), respectively. The observed electron density clearly stands in the map out as dark-blue regions. Difference density maps for BCDD@BUT-17 after removing the BCDD molecule: **c** 2D map calculated in the molecular plane supposed for BCDD (stick model of the BCDD is overlaid on a map), **d** 3D F_obs_–F_calc_ wire map with outlined negative (red) and positive regions of electron density. All maps were plotted at 0.10 Å grid resolution.

We then removed the BCDD from the reported BCDD@BUT-17 and refined a guest-free model. The agreement indices are nearly identical to those for the model including the guest molecule (R_1_ = 5.66% versus 5.62%, respectively). Moreover, the electron density difference map (Fourier transform of F_obs_–F_calc_) in the plane of BCDD (Fig. 1c) does not reveal peaks at the positions where the chlorine atoms are expected (which should clearly emerge, if the molecule was there). Only isolated electron density residuals are visible with peaks of at most 0.4 eÅ^−3^ height, just above a random error level, possibly associated with traces of disordered solvent molecules of second-row elements^4,5^. The 3D F_obs_–F_calc_ map reveals that these residuals occur outside the molecular plane of the hypothetical BCDD (Fig. 1d), but they are much smaller than the residuals inside the framework structure itself, due to minor deviations of the measured intensities from those predicted by the model. We used the routine SQUEEZE in Platon^6^ to compute the total amount of electron density not accounted by the model without BCDD. This integrates to just 3 electrons in the channels of the entire unit cell, where one would expect 6 molecules and therefore 768 electrons overall (assuming an occupation of 100%). Therefore, the XRD data do not support the claim that BCDD molecules have been adsorbed in the MOF. Possible confusion on the (low) content of molecules in the channels arises from the presence of some solvent (likely water) molecules. In fact, even though the impregnation procedure did not include aqueous solutions, some water molecules have been included by the authors in the BCDD@BUT-17 model.

Our finding contradicts the reported BUT-17 high removing efficiency towards BCDD (73%). We note however, that sample preparation and experimental conditions differed for the XRD and fluorescence measurements. Therefore, we cannot exclude (nor confirm) that BCDD is responsible for the fluorescence quenching.

Despite our scepticism, one should not conclude that XRD is unable to identify guest molecules present in traces inside pores of MOFs. This is achievable, but not straightforward. One problem is that guest molecules are often anchored by weak non-covalent interactions, much more flexible than the covalent ones in the framework, and therefore more prone to induce positional and dynamical disorder. Experimental procedures, such as rapid-cooling during the X-ray diffraction experiment may further aggravate the problem of disorder^7^ leading to a blurring of, especially, the high-resolution diffraction and resulting in smeared-out electron densities computed at the sites occupied by the guests. This may hamper the identification of the guest molecule and a sensible refinement of its molecular geometry.

However, the chemical and structural flexibility of MOFs has been successfully applied to design frameworks that avoid the disorder of the guest molecules. The coordinative alignment in the pores, for example, can be exploited for the determination of single-crystal X-ray structures, and even absolute configurations, of molecules, for which the structures cannot be solved from single crystals of the pure substance^4,8^. To make proper use of XRD, one should remember that the framework atoms contribute much more to the Bragg diffraction intensities than the disordered guest molecules. Therefore, only excellent crystal and data quality may enable a satisfactory recognition and modelling of the guest molecules. If this is not the case, the XRD data quality might still allow for modelling the framework structure but not more than that. Ramadhar et al.^9^. provide a solid overview of guidelines for the structure determination of guests inside MOFs.

In conclusion, the diffraction data in the article by Wang B. et al. does not show any evidence of BCDD absorbed in the MOF. XRD is an important technique capable of detecting and determining guests in MOF pores (qualitatively and quantitatively), but it can only succeed with excellent crystal and XRD data quality and when using great care during the modelling.

We want to stress that the reported crystal structure must arise from the data, not the data be interpreted based on a presumed structure without any other evidence. The analysis of electron density Fourier maps is the first and essential step in monitoring the correctness of the model. Instead, the use of constraints and restraints during the model refinement must be clearly justified and applied only after having unambiguously identified the elusive molecule(s) inside the pores.

We invite authors, referees, and editors to be more critical about the possibilities (which are many) and limitations (that are unavoidable) provided by XRD, in order to avoid reasoning that is not fully supported by the data.

## Data Availability

All data generated or analysed during this study are included in the published article in ref. 1. All relevant processed data are available from the authors upon reasonable request.
